# Diagnostic accuracy of damage-associated molecular patterns (DAMPs) in patients with heart failure with a reduced ejection fraction

**DOI:** 10.1017/cts.2017.11

**Published:** 2017-08-10

**Authors:** Justin Hartupee, Rohan Patel, Lora Staloch, Victor G. Dávila-Román, Lisa de las Fuentes, Eric Novak, Douglas L. Mann

**Affiliations:** Department of Medicine, Center for Cardiovascular Research, Cardiovascular Division, Washington University School of Medicine, St. Louis, MO, USA

To the Editor

Elevated levels of inflammatory mediators have been identified in patients with heart failure (HF) with a reduced ejection fraction (HFrEF) in direct relation to worsening New York Heart Association (NYHA) class [1]. However, the mechanism(s) responsible for elevated levels of inflammatory mediators in HF is not known. Recent studies have shown that dying cells release “damage-associated molecular patterns” (DAMPs) that provoke inflammatory responses through engagement of innate immune receptors (e.g., toll-like receptor 4) [2]. Accordingly we sought to determine whether DAMPs were elevated in HF patients in relation to NYHA class, as well as assess their diagnostic accuracy for HF. We examined the DAMP galectin-3 [3] which has a US Food and Drug Administration-approved assay for the diagnosis of HF, as well as two other DAMPs that have been linked to inflammation: calprotectin [S100A8/S100A9] and high mobility box group box 1 (HMGB1). NT-proBNP was used as the reference standard for diagnosing HF, and circulating levels of troponin T were used as a sensitive marker of myocyte injury in HF.

We measured circulating levels of the biomarkers in 120 controls (EF>55%) and 120 cases with NYHA class I–IV HFrEF (EF<40%) from the Washington University Heart Failure registry [4] who were matched on age, gender, diabetes, and hypertension. Measurement of NT-proBNP and high-sensitivity (hs) troponin T were measured using a commercial assay (Roche Diagnostics) and galectin-3 (R&D Systems), calprotectin (Hycult Biotech), and HMGB1 (IBL International) were measured by ELISA.

**Fig. 1:**
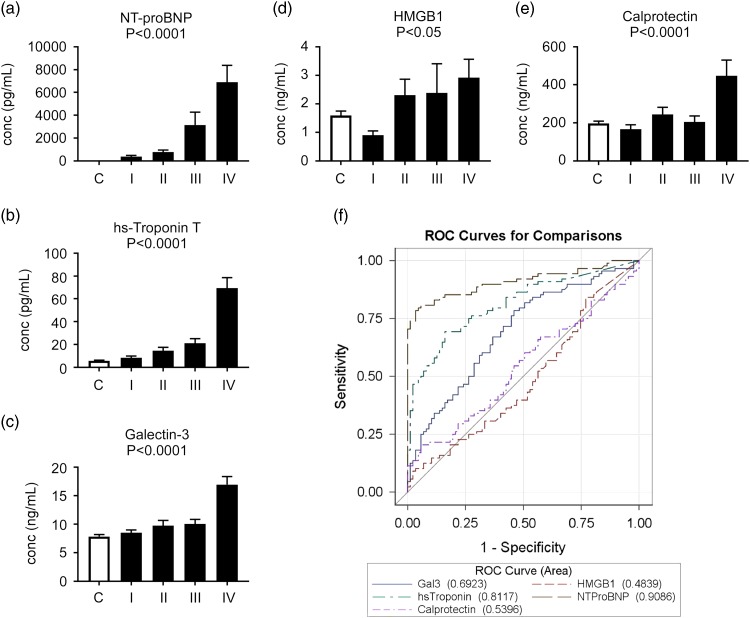
Damage-associated molecular patterns in heart failure (HF) with reduced ejection fraction (EF). (*a*–*e*) Biomarker levels in controls (C) and patients with New York Heart Association class I–IV HF for NT-proBNP (*a*), high-sensitivity (hs) troponin (*b*), galectin-3 (*c*), HMGB1 (*d*), and calprotectin (*e*). Data expressed as mean and error bars represent SEM. *p* Values determined by 1-way analysis of variance. (*f*) The ability of different biomarkers to discriminate HF from controls was evaluated by determining the area under the Receiver Operating Characteristic (ROC) curve. The area under the curve ranges from 0 to 1 and describes accuracy, with 1 identifying perfect predictive ability and 0.5 indicating that performance is no better than chance.

Fig. 1 shows that circulating levels of NT-proBNP (a), hs-troponin T (b), and galectin-3 (c) increased with worsening NYHA class; whereas levels of HMGB1 (d) were elevated similarly in NYHA class II–IV HF and levels of calprotectin (e) were only elevated in NYHA class IV HFrEF. We next assessed Receiver Operating Characteristics curves for each biomarker to determine their accuracy to discriminate HFrEF from controls (f). As shown in Table 1, the rank order for diagnostic accuracy was NT-proBNP>hs-troponin T>galectin-3>calprotectin>HMGB1. The diagnostic accuracy of NT-proBNP was significantly better than any of the biomarkers studied [5]. Interesting, neither calprotectin nor HMGB1 were accurate in terms of diagnosing patients with HFrEF.

**Table 1:** Diagnostic accuracy of biomakers assessing area under curve (AUC)

Predictor	AUC	95% CI	*p*-value	*p*-value vs. NT-proBNP
NT-proBNP	0.91	(0.86, 0.96)	<0.001	–
hs-Troponin T	0.81	(0.75, 0.88)	<0.001	0.002
Galectin-3	0.69	(0.61, 0.77)	<0.001	<0.001
Calprotectin	0.54	(0.45, 0.63)	0.37	<0.001
HMGB1	0.48	(0.40, 0.57)	0.71	<0.001

CI, confidence intervals; HMGB1, high mobility group box 1; hs, high sensitivity.

We conclude that although classical DAMPs such as HMGB1 and calprotectin are elevated in HFrEF patients, they do not track with NYHA class, and therefore are not likely to explain the increased levels of inflammatory mediators observed in HF, nor are these DAMPs as accurate for diagnosing HF as traditional markers of cell injury, such as troponin T.
